# Virtual reality cue-exposure therapy in reducing cocaine craving: the Promoting Innovative COgnitive behavioral therapy for Cocaine use disorder (PICOC) study protocol for a randomized controlled trial

**DOI:** 10.1186/s13063-024-08275-7

**Published:** 2024-06-27

**Authors:** T. Lehoux, Antonio Capobianco, Jérôme Lacoste, Sloane Rollier, Yann Mopsus, Manuela Melgire, Flavien Lecuyer, Miguel Gervilla, Luisa Weiner

**Affiliations:** 1 Laboratoire de Psychologie Des Cognitions, University of Strasbourg, 4 Rue Blaise Pascal, 67081 Strasbourg, France; 2 Laboratoire ICube – Equipe IGG, University of Strasbourg, 300 Boulevard Sébastien Brant, 67412 Illkirch, France; 3 Service d’Addictologie, University Hospital of Martinique - Hôpital Pierre Zobda-Quitman (CHU de Martinique), CS 90632 - 97261 Fort-de-France Martinique, France; 4 Research Methodological Support (USMR), Délégation à La Recherche Clinique et à L’Innovation, University Hospital of Martinique - Hôpital Pierre Zobda-Quitman (CHU de Martinique), CS 90632 - 97261 Fort-de-France, Martinique, France; 5 Department of Addictology, CSAPA Saint-Esprit, Saint-Esprit Hospital (CH de Saint-Esprit), BP 176 Route du Petit-Bourg, 97270 Saint-Esprit, Martinique, France

**Keywords:** Virtual reality cue-exposure therapy (VRCET), Cue-exposure therapy (CET), Cocaine use disorder, Cocaine craving, Memory-focused cognitive therapy (MFCT), Cognitive behavioral therapy

## Abstract

**Background:**

Cocaine craving is a central symptom of cocaine use disorders (CUD). Virtual reality cue-exposure therapy for craving (VRCET) allows more immersive, realistic, and controllable exposure than traditional non-VR cue-exposure therapy (CET), whose efficacy is limited in treating substance use disorders. The purpose of this study is to evaluate the efficacy and acceptability of VRCET, as a stand-alone and add-on intervention (i.e., combined with cognitive therapy), compared to a picture-based CET (PCET), in reducing self-reported cocaine craving in inpatients hospitalized for CUD.

**Methods:**

Fifty-four inpatients hospitalized for CUD will be randomized in one of two intensive 3-week treatment arms: 10 meetings/2-week treatment of VRCET plus 5 meetings/1-week treatment of memory-focused cognitive therapy (MFCT; experimental arm), or 15 meetings/3-week treatment of PCET (active control arm). The Craving Experience Questionnaire (CEQ – F & S) will be used to assess the primary outcome, i.e., the post-treatment decrease of self-reported cocaine craving frequency (within the past 2 weeks) and intensity scores (in VR exposure to cocaine cues). Secondary endpoints include urinary, physiological, and self-reported cocaine use-related measures. Assessments are scheduled at pretreatment, after 2 weeks of treatment (i.e., VRCET vs. PCET), post-treatment (3 weeks, i.e., VRCET + MFCT vs. PCET), and at 1-month follow-up. Acceptability will be evaluated via (i) the Spatial Presence for Immersive Environments – Cybersickness along VRCET and (ii) the Client Satisfaction Questionnaires after 2 weeks of treatment and post-treatment.

**Discussion:**

This study will be the first to evaluate the acceptability and efficacy of VRCET for CUD, as a psychotherapeutic add-on, to reduce both cocaine craving frequency and intensity. Additionally, this study will provide evidence about the specific interest of VRCET, compared to a non-VR-based CET, as a cue reactivity and exposure paradigm for treating substance use disorders.

**Trial registration:**

NCT05833529 [clinicaltrials.gov]. Prospectively registered on April 17, 2023.

## Administrative information

Note: the numbers in curly brackets in this protocol refer to SPIRIT checklist item numbers. The order of the items has been modified to group similar items (see http://www.equator-network.org/reporting-guidelines/spirit-2013-statement-defining-standard-protocol-items-for-clinical-trials/).

**Table:** 

Title {1}	SPIRIT guidance: Descriptive title identifying the study design, population, interventions, and, if applicable, trial acronym. Virtual reality cue exposure therapy in reducing cocaine craving: the Promoting Innovative COgnitive-behavioral therapy for Cocaine use disorder (PICOC) study protocol for a randomized controlled trial.
Trial registration {2a and 2b}.	SPIRIT guidance: Trial identifier and registry name. If not yet registered, name of intended registry. Item 2b is met if the register used for registration collects all items from the World Health Organization Trial Registration Data Set. NCT05833529 [ClinicalTrials.gov]. Prospectively registered on April 17, 2023.
Protocol version {3}	SPIRIT guidance: Date and version identifier. Version 01 of 27–05-2024.
Funding {4}	SPIRIT guidance: Sources and types of financial, material, and other support. This study has been published under the framework of the Idex University of Strasbourg and has been supported by the French government funding managed by the National Research Agency under the Investments for the Future program (PIA) with the grant ANR-21-ESRE-0030 (CONTINUUM project) and the French National Cancer Institute (INCa; SPAV1-22–023) as well as the Public Health Research Institute (IReSP), as part of the Thomas Lehoux’s doctoral funding SPADOC21 (#AAC21-SPA-04).This study was supported by a grant from the Groupement Interrégional de Recherche Clinique et d’Innovation Sud-Ouest Outre-Mer Hospitalier (APIDOM 2022).
Author details {5a}	SPIRIT guidance: Affiliations of protocol contributors. (First and corresponding author) T. Lehoux: Laboratoire de Psychologie des Cognitions (University of Strasbourg), France A. Capobianco: Laboratoire ICube – Equipe IGG (University of Strasbourg), France J. Lacoste: Service d’Addictologie (CHU de Martinique), France S. Rollier: Délégation à la Recherche Clinique et à l’Innovation (CHU de Martinique), France M. Melgire: CSAPA Saint-Esprit (CH de Saint-Esprit), France Y. Mopsus: Délégation à la Recherche Clinique et à l’Innovation (CHU de Martinique), France F. Lecuyer: Laboratoire ICube – Equipe IGG (University of Strasbourg), France M. Gervilla: Laboratoire ICube – Equipe IGG (University of Strasbourg), France L. Weiner: Laboratoire de Psychologie des Cognitions (University of Strasbourg), France
Name and contact information for the trial sponsor {5b}	SPIRIT guidance: Name and contact information for the trial sponsor. University Hospital of Martinique (UHM) Direction Générale – Hôpital Pierre Zobda Quitman Martinique, 97261 Fort-de-France Cedex direction.generale@chu-martinique.fr
Role of sponsor {5c}	SPIRIT guidance: Role of study sponsor and funders, if any, in study design; collection, management, analysis, and interpretation of data; writing of the report; and the decision to submit the report for publication, including whether they will have ultimate authority over any of these activities. The study sponsor (i.e., University Hospital of Martinique) and funders played no role in the design of the study; collection, analysis, and interpretation of the data; and writing the manuscript.

## Introduction

### Background and rationale {6a}

Cocaine is the second most widely used illicit substance in Europe: its annual prevalence of use reached 1.6% of the French population in 2017 and is sharply superior in overseas regions compared to continental European territories [1, 2]. Moreover, one in 6 cocaine users will suffer from cocaine use disorder (CUD; [3]), with significant impacts on health and well-being [4, 5]. The annual social cost of CUD is high as it is estimated to be of 45,469 G$ in the United States alone [6].

Cocaine craving is considered as a central DSM-5 (Diagnostic and Statistical Manual of Mental Disorders – 5th version; [7]) CUD symptom that can be defined as an obsessive motivation to use, which can be automatically elicited when exposed to cocaine cues [8–10]. Importantly, a meta-analysis of 41 cue-reactivity studies suggests that cocaine craving might be the most intense across substances [11]. Hence, craving-focused therapeutic interventions might be of clinical interest for treating CUD, since cocaine craving is a significant proxy of cocaine use and predicts relapse until 3 months post-treatment [12, 13].

Cognitive behavioral therapy (CBT) for substance use disorders targets, *inter alia*, cognitive, affective, and situational triggers for substance use and is shown to be potentially efficacious in treating CUD (*N* = 13 studies; [14]). Stemming from CBT, cue-exposure therapies (CET) essentially aim to extinguish drug cues-associated responses, e.g., cue-induced craving, through repeated non-reinforced exposures. Nevertheless, CET have shown non-significant to small treatment effects in treating SUD according to meta-analytic evidence [15, 16]. These unfavorable results might be due to cue-exposure parameters that have yet to be fully considered when providing CET [15]. For instance, cue-reactivity research suggests the interest of providing more multisensorial, realistic, and ecologically valid exposure environments for improving exposure to substance cues and inducing higher levels of craving [17]. Thus, improving cue-exposure might be of promising value for CET since craving response to substance cues moderates craving reduction [18] and predicts substance use latency, dependence severity and withdrawal reinstatement in a CET context [19].

Virtual reality (VR) refers to technological devices allowing immersive, interactive and multisensorial exposure to fully computer designed three-dimensional (3D) real-life or imaginary environments (for a systematic review, see: [20]). There is a consensus on the fact that virtual reality exposure (VRE) features might have the potential to improve CET for SUD, allowing controlled exposure to various and realistic contextual substance cues (e.g., handling crack pipes in the presence of peers, self-injecting cocaine), which could not be used in vivo for safety purposes (for a systematic review, see: [21]). This might be of particular interest for generalizing CET effects and preventing cue-induced craving rebound (e.g., renewal effect) in patients with CUD, as cocaine cue reactivity strongly predicts relapse post-treatment [22, 23]. In addition, VR high-immersive properties enhances the feeling of being present in an exposure environment (i.e., sense of presence; [24]), which encompasses its believability, realism and naturalness (i.e., ecological validity; [25]). Interestingly, in one study, ecological validity in VRE was found to moderate and enhance craving induction in heavy drinkers [26]. Finally, numerous studies have demonstrated that VRE to substance cues might be feasible and capable to induce craving response in nicotine, alcohol, but also in cocaine users (for a systematic review: [27–29]). Consequently, VRE to substance cues might be of relevant interest in addictology settings for diagnostic, prognostic and treatment purposes (for a systematic review, see: [30]). For instance, VRE cue-induced craving has been shown to be a significant proxy of drinking status [31], nicotine [32], or alcohol dependence severity [26], as well as treatment response [33]. Further, several VR-based CET studies in SUD found significant associations between virtual reality cue-exposure therapy (VRCET) and post-treatment reduction in smoked cigarettes/day, air expired CO_2_, background, and VRE-induced craving, as well as an increased abstinence and readiness to quit using [34, 35].

However, VRCET in these studies was either used as an add-on to an active treatment condition, or not compared to a control condition [30], which does not provide further insights into the clinical benefit or risk associated to VRCET over classical CET or CBT. For instance, some evidence suggest that VRCET, and CET more broadly, may increase the risk of relapse among patients treated for nicotine use disorder (NUD), compared to CBT alone [36]. Accordingly, therapeutic interventions for SUD which promote effective coping with lapses are suggested to be the most useful supplement to CET [15]. In line with this, in patients with CUD, positive relationships are shown to exist between beliefs about abstinence self-efficacy, lower urges in high-risk situations and cocaine use up to 3 months post-treatment [37]. Interestingly, memory-focused therapy cognitive therapy (MFCT) is a novel and promising structured CBT around, inter alia, patient’s reliving, cognitive restructure and restructure of sensory images, negative appraisals, and complex emotional responses associated with consolidated cocaine-related memories, as well as the use of general relapse prevention skills [38], and whose the preliminary efficacy in reducing cocaine use and craving was demonstrated in an external RCT on outpatients treated for CUD [39, 40]. Suggested as an adjunctive CBT intervention for CUD [39], MFCT might be of substantial interest for enhancing therapeutic response and preventing risk of relapse of patients involved in our VRCET-based experimental therapeutic arm. Consequently, we decided to combine our VRCET intervention phase with a consecutive MFCT phase, specifically intended to enhance the overall patient’s control over their cocaine craving-related dysfunctional thoughts, emotions, and behaviors up to 3-month post-treatment [39]. Finally, given meta-analytic evidence suggesting that brief therapy formats might enhance the overall effect of CBT for SUD (*N* = 47 studies; [14], both our experimental and control therapeutic arms will be delivered in a brief (3 weeks) and daily (5 days/week) format, which has been shown to be feasible safe and acceptable in outpatients with CUD [39].

Hence, our experimental therapeutic arm will consist of 2 consecutive weeks of daily-delivered VRCET, followed by 1 week of daily-delivered MFCT, whereas the active comparator arm will consist of 3 consecutive weeks of daily-delivered PCET.

### Objectives {7}

The primary objective of our parallel randomized controlled trial is to evaluate the efficacy and acceptability of an intensive 3-week VRCET-based psychotherapy (VRCET +), i.e., VRCET + MFCT, in reducing self-reported cocaine craving in inpatients hospitalized for CUD, by comparing its immediate post-treatment effects to the ones of a traditional PCET.

The secondary objectives of our trial will be:

1. To evaluate the effects after 2 weeks of treatment (V1; VRCET vs. CET), post-treatment (V2; VRCET + vs. PCET), and 1-month post-treatment (V3; VRCET + vs. PCET) on urinary, physiological, and self-reported measures of cocaine use-related measures (abstinence, risk of relapse, self-efficacy to cope with craving, emotional dysregulation, and identification to dysfunctional thoughts)
2. To evaluate the effects of VRE to cocaine cues on cue-specific cocaine craving-related measures (cocaine craving, emotional states, self-efficacy to cope with craving) and cue-exposure specific measures (sense of presence, ecological validity, cybersickness) compared to a traditional non-VR pictures-based cue-exposure (PCE).
3. To evaluate the cognitive, behavioral, or emotional factors (cocaine craving, self-efficacy to cope with craving, dysfunctional thoughts, cocaine use and emotional dysregulation) mediating the efficacy of VRCET + in decreasing self-reported cocaine craving.
4. To evaluate the acceptability of (i) our intensive VRCET as a stand-alone (V1) or add-on (V2; VRCET +) therapeutic intervention via client satisfaction towards psychotherapeutic services measures and (ii) VRE, as a cue-exposure paradigm via cybersickness self-reported measures.

### Trial design {8}

This project is a two-center parallel, randomized, controlled, superiority trial, with a 1:1 allocation ration and two 3-week treatment arms (15 meetings): a VRCET-based experimental arm (VRCET + ; 2 week VRCET + 1 week MFCT) and a PCET-based active control arm.

## Methods: participants, interventions, and outcomes

### Study setting {9}

The PICOC trial will be conducted in 2 French overseas national centers (Martinique University Hospital and Saint-Esprit Hospital, Martinique, France). Eligible patients will be recruited and hospitalized in the 2 addictology inpatient residential services, where data will be collected during the trial. The Martinique University Hospital (UMH) is the largest French and English-speaking Caribbean University Hospital. In addition, the UMH is a 1600-bed institution, including 680 medical, 273 surgical, obstetrics, and 30 intensive care unit beds. Both the UMH and Saint-Esprit Hospital are specialized in treating SUDs, offering out and residential hospitalization services.

### Participants

#### Eligibility criteria {10}

##### Inclusion criteria

Patients must meet the following criteria to be eligible for the study:

- Patient with a CUD (SCID-5 CV; [41]) and treated as an inpatient at one of the two investigation centers (i.e., Martinique University Hospital or Saint-Esprit Hospital)
- URICA score indicating “Action” or “Maintenance” readiness to stop using cocaine [42]
- Patient affiliated to a social health insurance plan
- Patient ≥ 18 years of age

##### Non-inclusion criteria

If the patients meet any of the following criteria at the screening visit, they will not be eligible for the study:

- Patient in current high suicidal risk, post-traumatic stress, psychotic, mania, or hypomania episode according to their M.I.N.I.5. score (DSM-IV; [43])
- Spatial Presence in Immersive Environments – Cybersickness score indicating cybersickness symptoms using VR (≥ 7), as assessed during a tutorial VRE task at inclusion visit [44]
- Patient with current medical condition (e.g., cardiac or blindness) at risk for safety or compliance in protocol, as assessed by one of the investigator physicians
- Patient care under constraint or patient deprived of freedom because of a judicial measure
- Patient who does not speak and read French

##### Inclusion criteria for individuals who will perform the interventions

- The facilitators of both psychotherapies will be mental health professionals (physician or psychologist) trained in delivering CET, VRCET, MFCT, and CBT

#### Who will take informed consent? {26a}

Patients diagnosed for CUD and treated as inpatients at one of the investigation centers residential addictology unity will be invited to meet with the research psychiatrist or psychologist for receiving study information. During this meeting, patients willing to participate will be invited to the screening meeting for eligibility assessment and signing the informed consent.

#### Additional consent provisions for collection and use of participant data and biological specimens {26b}

This trial does not aim to collect and use participant data and biological specimens in ancillary studies.

### Interventions

#### Explanation for the choice of comparators {6b}

The active control group will consist of an intensive 3-week PCET. We will control for non-specific and/or treatment-specific processes known to affect target outcomes (e.g., research involvement, time, placebo, attention, information, and the effects of a competing treatment [45]). Consistently, recent systematic reviews and meta-analyses showed overall moderate additional effects of CET, compared to active control conditions in treating SUD [15, 16, 46].

#### Intervention description {11a}

Prior to the start of CET (week 1), both VRCET + and PCET intervention arms will be offered a single session psychoeducation on cocaine craving (e.g., its obsessive, cerebral and involuntary nature), CET principles (e.g., craving time-course and desensitization), and rules (e.g., repeated, prolonged, and diversified exposures; material available on request).

##### Experimental arm (VRCET +)

The experimental arm (VRCET +) will consist in an intensive 3-week treatment of 10 meetings of VRCET, followed by 5 meetings of MFCT. All meetings will last 90 min and will be delivered on 5 consecutive days, i.e., Monday to Friday. VRCET meetings will take place in a 2 consecutive weeks period (weeks 1 and 2). MFCT meetings will take place in the 1-week period following VRCET (week 3).

##### Virtual reality cue-exposure therapy (VRCET)—weeks 1 and 2

**Fig. 1:**
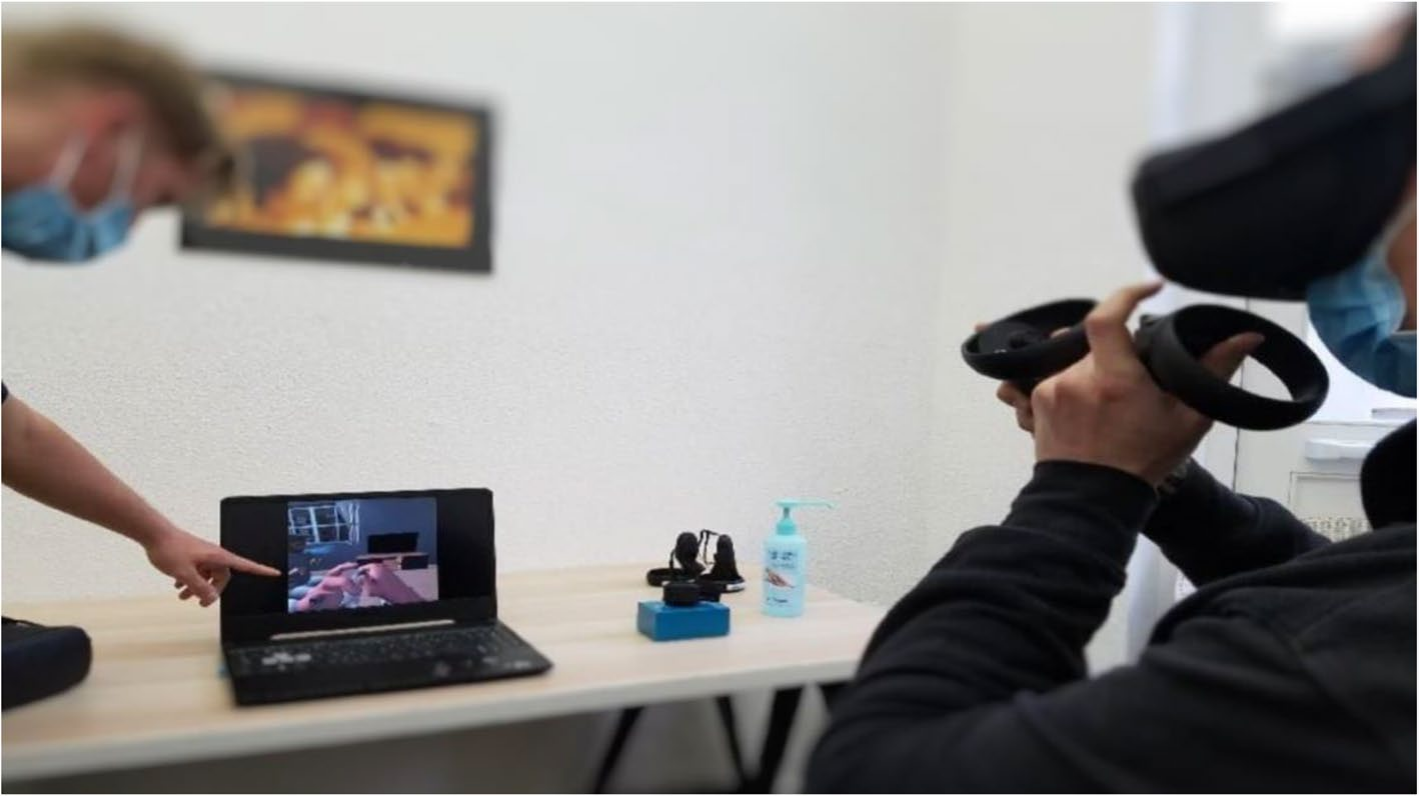
Fictive VRE methodological set-up

**Fig. 2:**
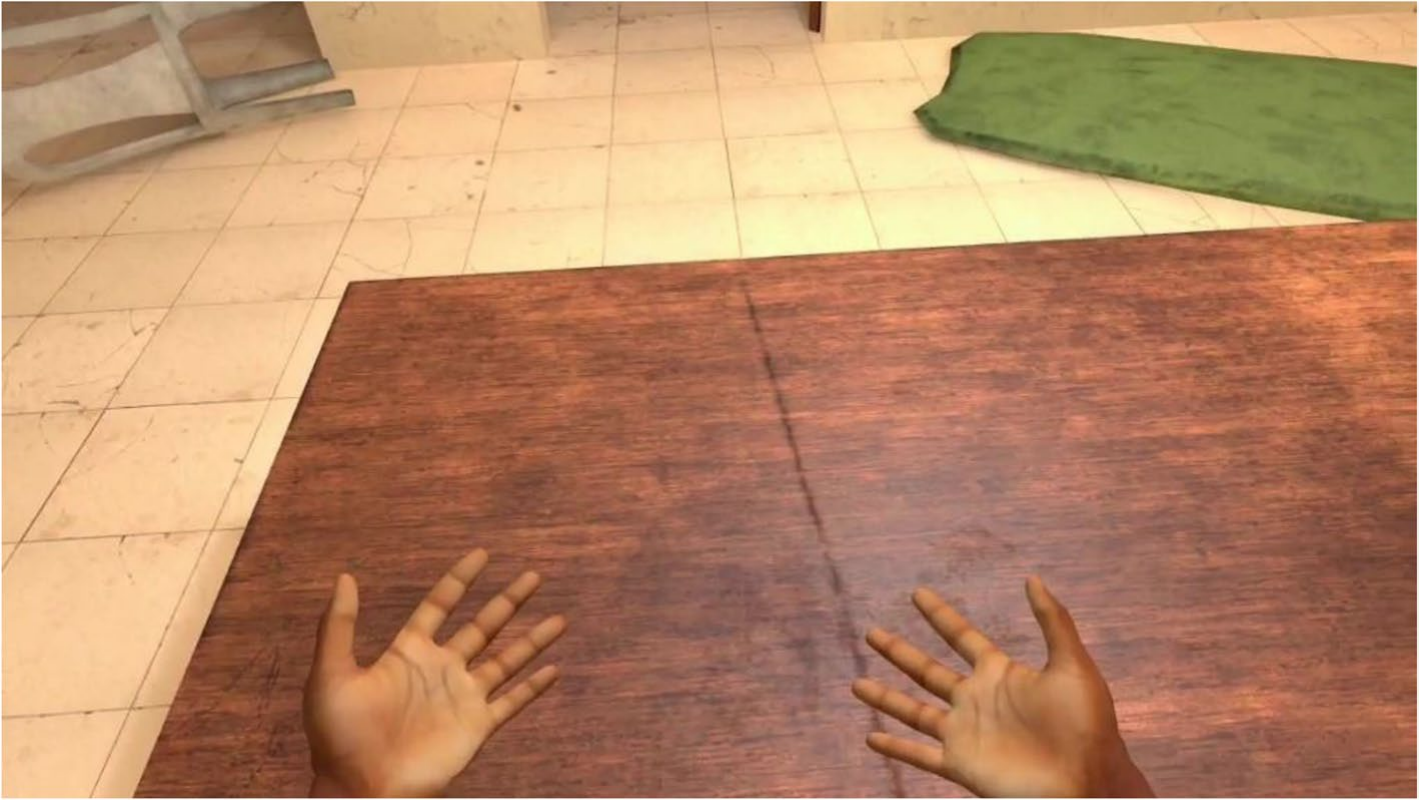
VRE 1st-person view with virtual controllable hands

VRCET will be performed using Meta Quest 2 VR headset. The VR application used in the present trial was designed by the ICube and LPC laboratories (Strasbourg University) in collaboration with the Saint-Esprit and Martinique Hospital Centers. The exposure setting is interactive, visually, and audio immersive (360° and first-person view). Participants will be able to virtually rotate and move their head, hands, and their upper body thanks to a 6 degrees of freedom headset/hand controllers’ system. Participants will remain seated on a 360°-rotating stool and will be able to move around in VR environments thanks to a laser-guided teleportation (Figs. 1 and 2). Finally, participants will see and use virtual hands to grasp, hold, and move virtual trivial or cocaine-related objects, as outlined in Table 1.

**Table 1:** Title and content of exposure situations per cocaine using mode across VRCET and PCET interventions (experimental and control arms)

**Common content across situations:** *I’m at home, listening to a vocal message from two peers about their imminent visit. After a short waiting-time, peers are coming by. They and I are sitting down at a living room table. They are talking about ways to get cocaine, cocaine use as well as its acute effects. (…)*
**Situation 1—Peers talking about cocaine:** *(…) There is neither cocaine nor related paraphernalia in this situation*
**Situation 2—Peers using cocaine:** *(…)* Whilst speaking, peers are taking cocaine and related paraphernalia out of their pocket and are setting right in front of them on the table. They are preparing and self-administrating cocaine doses. There is no neither cocaine nor related paraphernalia in front of me and I cannot hold any in this situation
**Situation 3—Holding cocaine while peers are using it:** *(…)* Whilst speaking, peers are taking cocaine and related paraphernalia out of their pocket and are setting right in front of them on the table. They are preparing and self-administrating cocaine doses. There are cocaine and relatedparaphernalia in front of me and I can hold some in hands. I cannot neither prepare nor self-administrate cocaine doses in this situation
**Situation 4—Preparing cocaine while peers are using it:** (…) Whilst speaking, peers are taking cocaine and related paraphernalia out of their pocket and are setting right in front of them on the table. They are preparing and self-administrating cocaine doses. There are cocaine and relatedparaphernalia in front of me and I can hold in hands and prepare some cocaine doses. I cannot self-administrate cocaine in this situation
**Situation 5 – Preparing cocaine and using it with peers:** (…) Whilst speaking, peers are taking cocaine and related paraphernalia out of their pocket and are setting right in front of them on the table. They are preparing and self-administrating cocaine doses. There are cocaine and relatedparaphernalia in front of me and I can hold in hands, prepare and self-administrate some cocaine doses in this situation
**For cocaine snorters** **Items available:** cocaine powder bag, sterile field, paper tube, card **Interaction:** pouring out cocaine on the field, making a cocaine line with the card, snorting the line with the paper tube
**For crack cocaine smokers** **Items available:** crack cocaine bag, sterile field, steel spoon, water pipe, baking soda (sodium bicarbonate), glass pipe, lighter **Interaction:** pouring out cocaine, water and baking soda into the spoon; heating the mix with the lighter; pouring out the crack cocaine rock into the glass pipe; heating the pipe with the lighter; inhaling crack smoke from the pipe
**For cocaine injectors** **Items available:** cocaine powder bag, sterile field, steel spoon, water pipes, syringe **Interaction:** pouring out cocaine and water into the spoon; filling up the syringe with diluted cocaine; self-injecting diluted cocaine into one’s arm

**Fig. 3:**
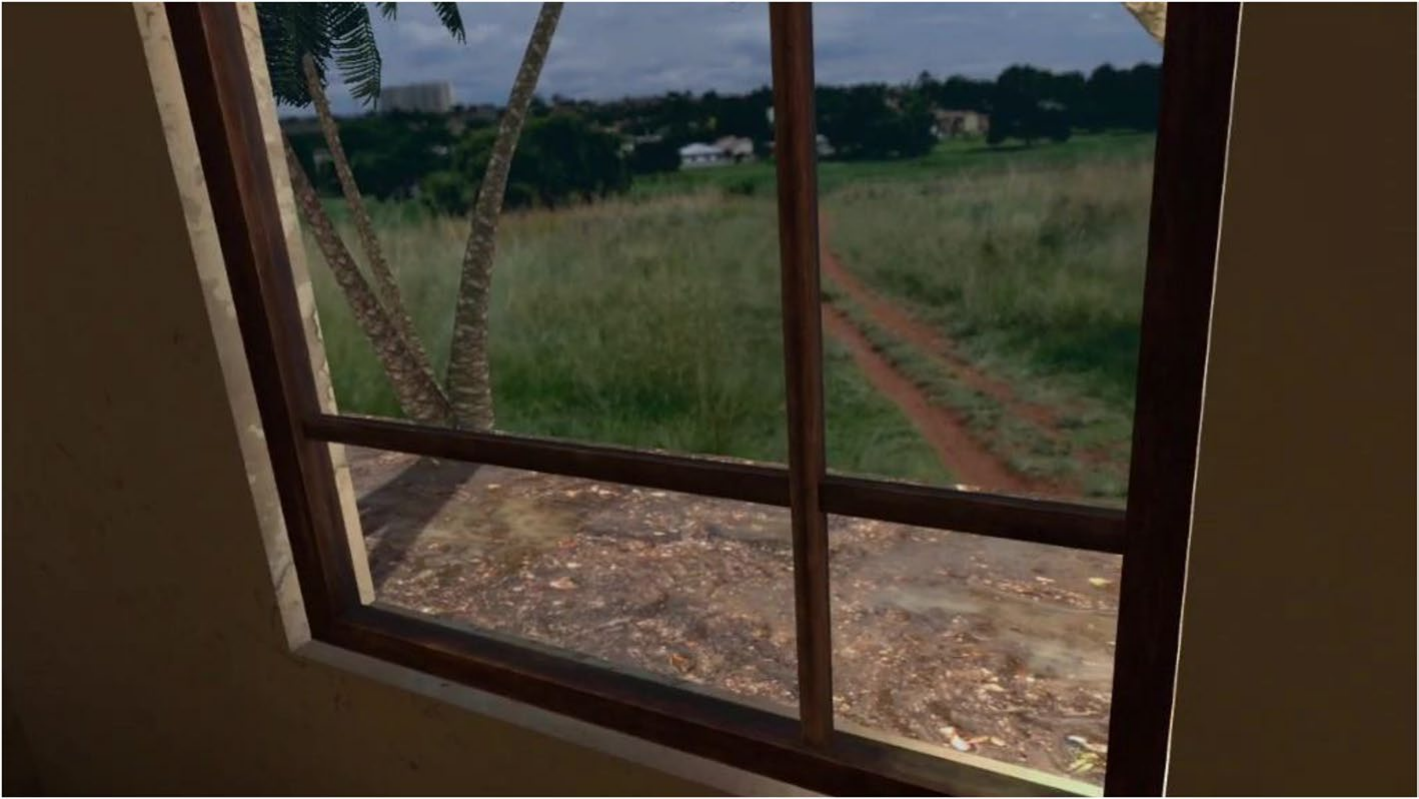
VRE window view on typical Martinican surroundings

**Fig. 4:**
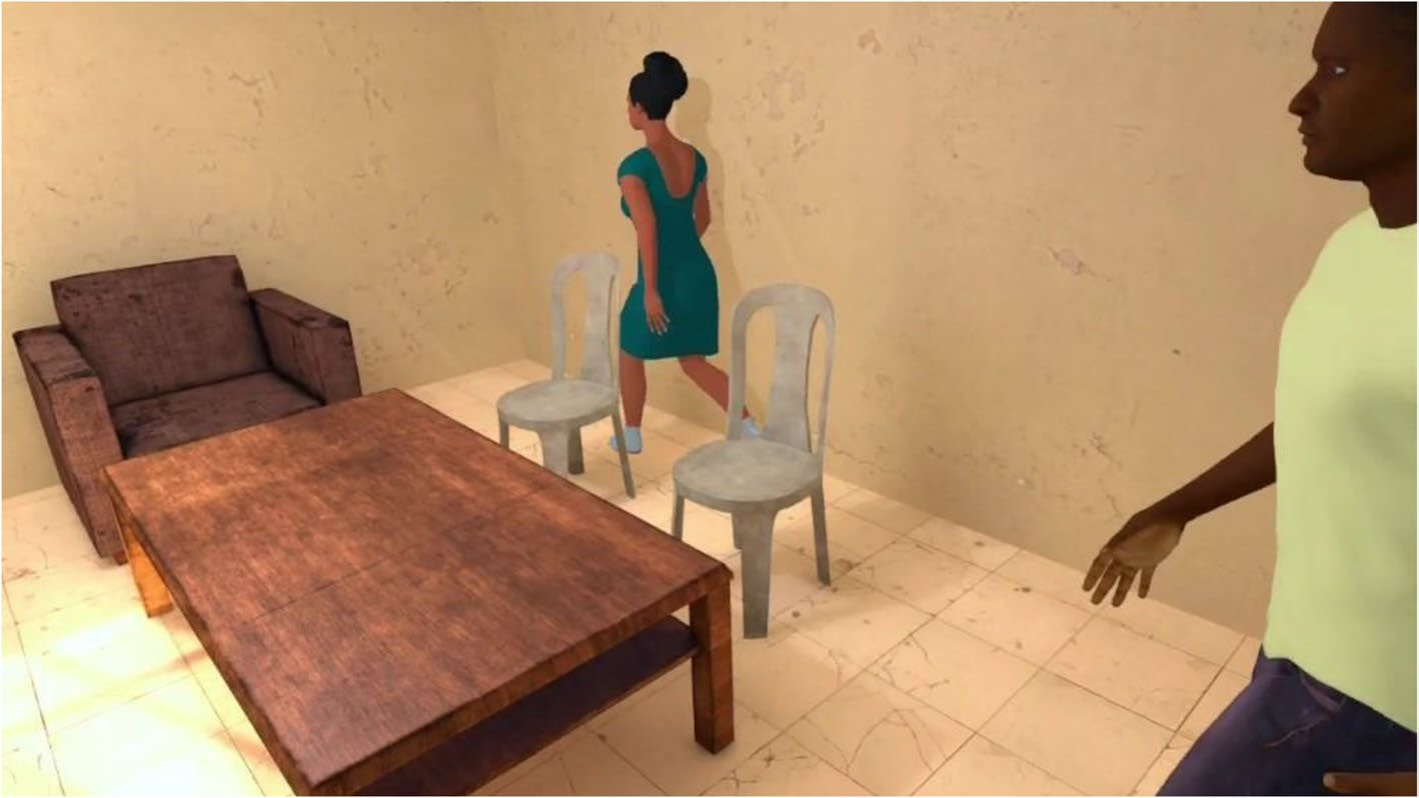
Virtual 1st-person view from VRCET and PCET Situation 1

**Fig. 5:**
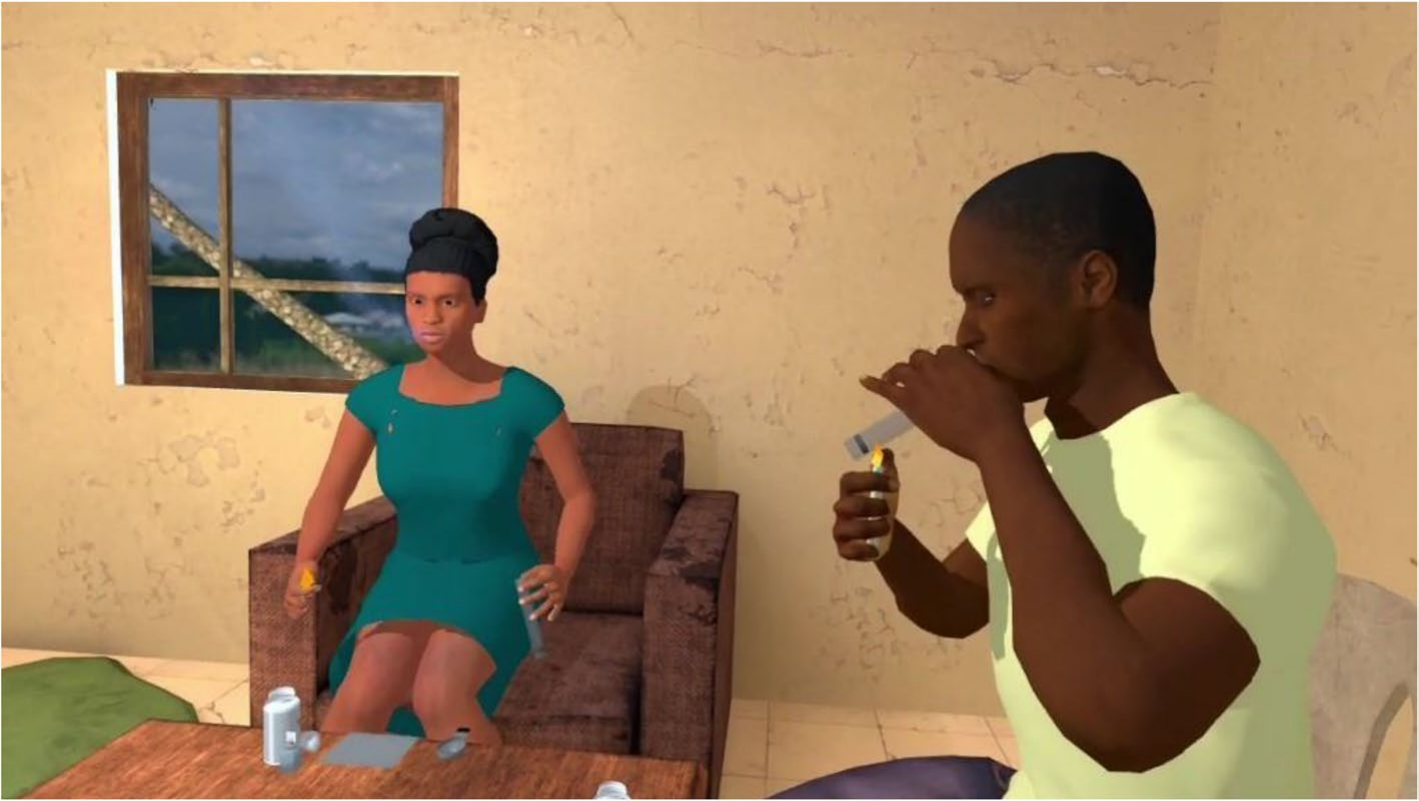
Virtual 1st-person view from VRCET and PCET Situation 2

**Fig. 6:**
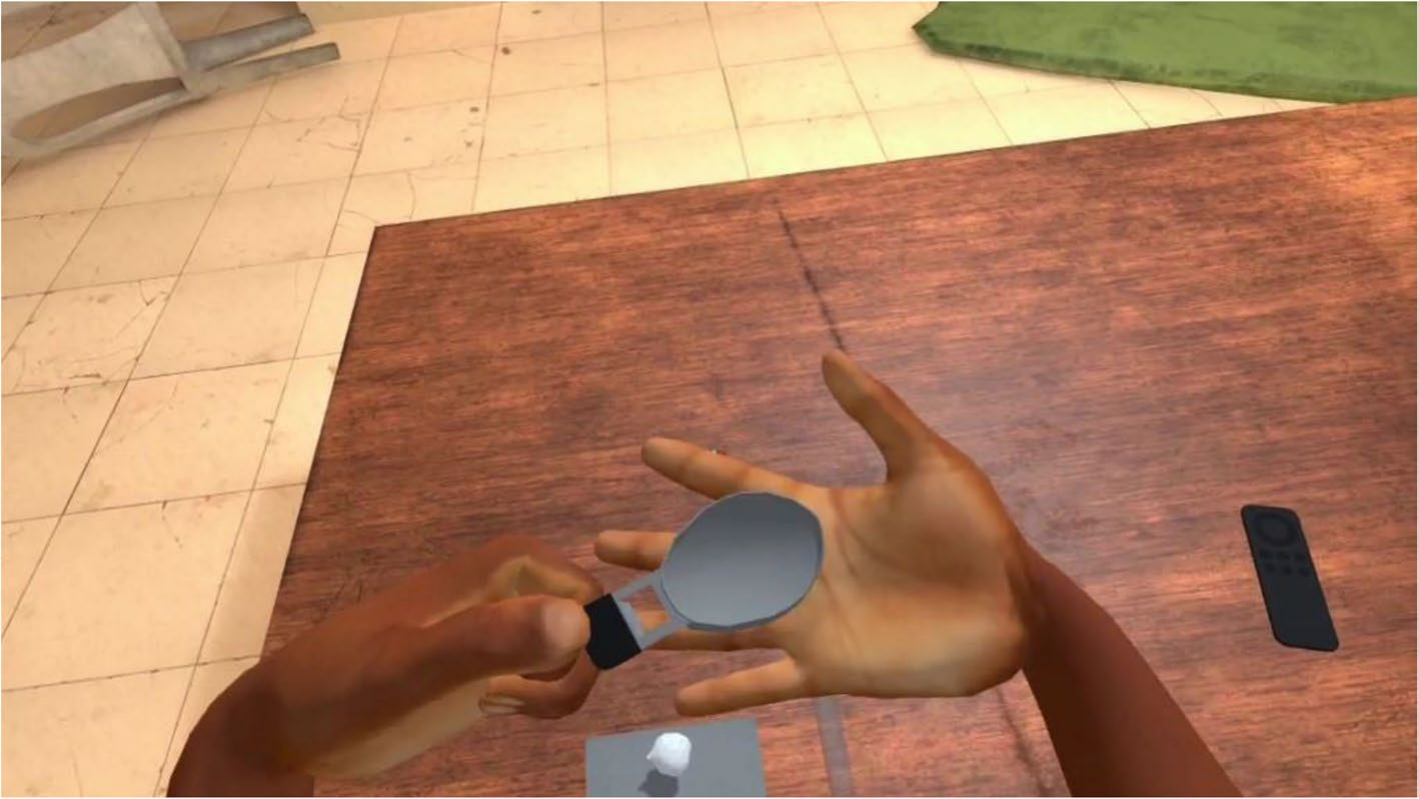
Virtual 1st-person view from VRCET and PCET Situation 3

**Fig. 7:**
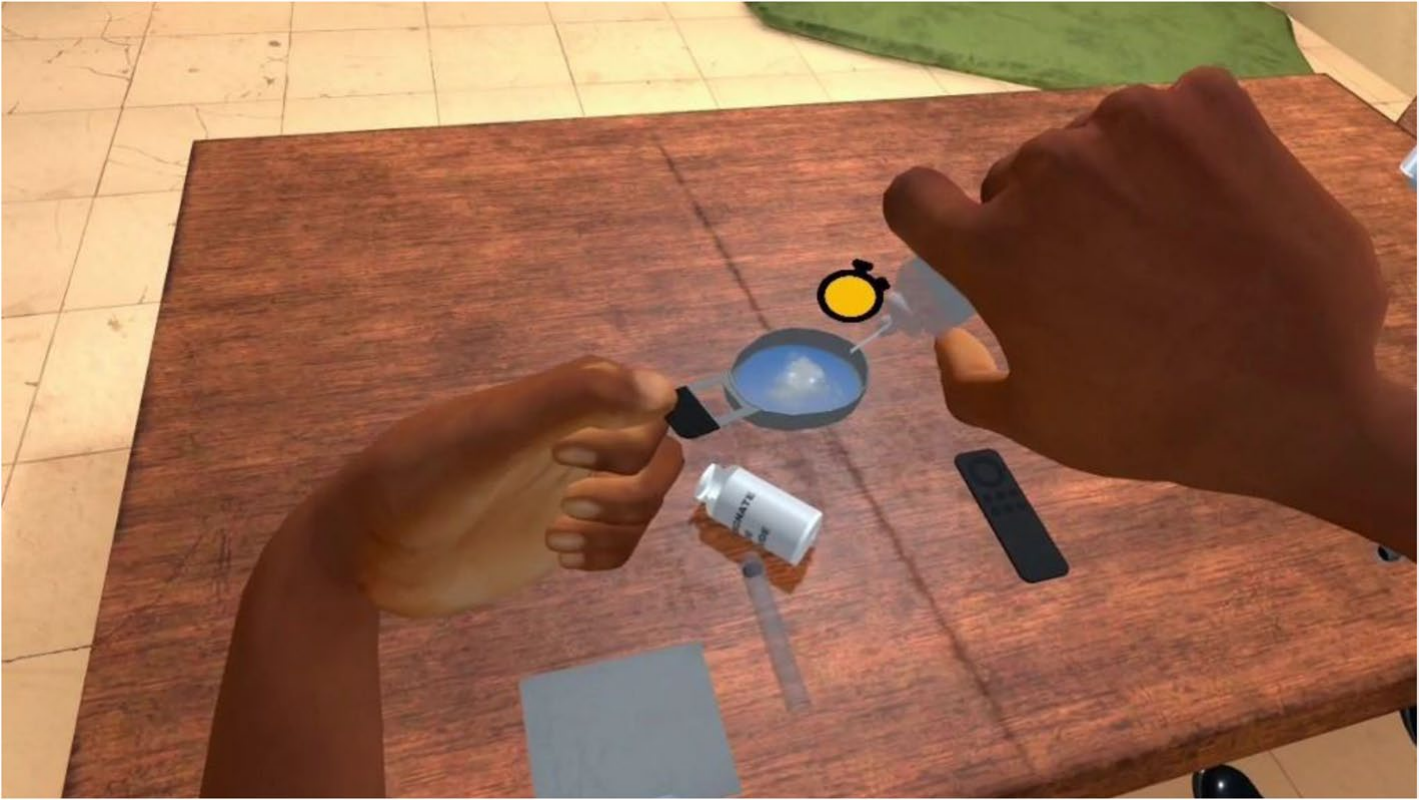
Virtual 1st-person view from VRCET and PCET Situation 4

**Fig. 8:**
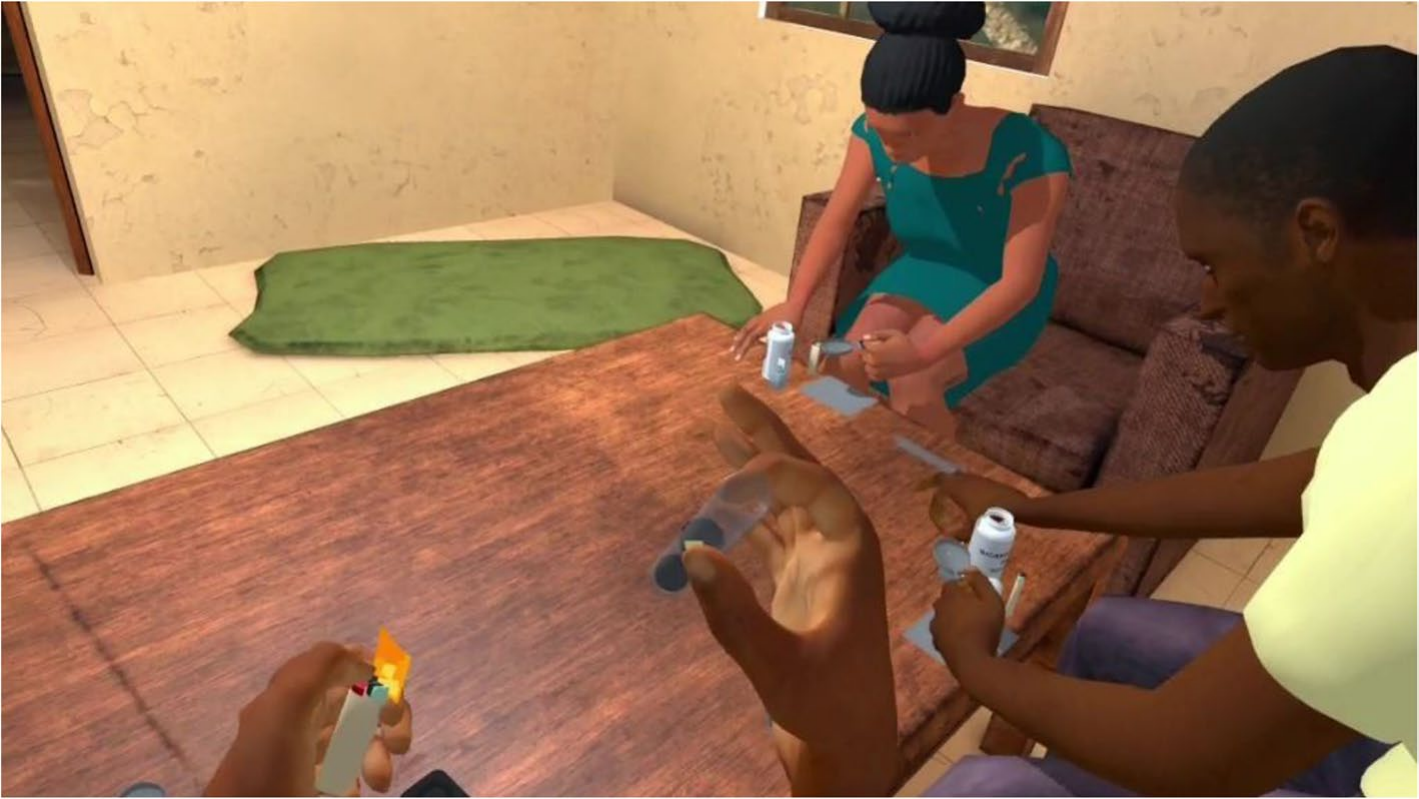
Virtual 1st-person view from VRCET and PCET Situation 5

The exposure in VRET will be habituation-focused, i.e., aiming to reduce the craving response to cocaine-related cues after its repeated, prolonged, and non-reinforced stimulation [47, 48]. To isolate the specific effect of virtual reality on this plausible desensitization mechanism underlying cue-exposure extinction, no other concurrent cognitive, behavioral, or emotional intervention (e.g., relaxation) will be delivered during VRE to cocaine-related cues. In addition, several methodological recommendations arising from meta-analysis of CET for SUDs will be applied to optimize our CET design [15]. First, in order to prevent post-treatment spontaneous craving recovery, i.e., the re-emerging of the extinguished craving response following passage of time between extinction to re-exposure to the cocaine-related cues, VR cue-exposures (VRCE) will be repeated and spaced both between and intra-VRCET sessions [15]. The VRCET phase will consist in a total of 40 repeated 10-min virtual reality exposures (VRE) to cocaine-related cues spread out on 10 daily VRCET sessions. Each VRCET session will consist of 4 VRCE with 2 different VRCET cocaine-related environments per session. Importantly, VRCE to the same cocaine-related environments will be spaced intra (30 min) and between (48 h) VRCE sessions. Secondly, in order to generalize the habituated response to the cocaine-related cues to contexts other than the treatment setting, and to prevent any renew of extinguished cue reactivity, VRCET will be conducted in multiple and various virtual cocaine-related contexts [23, 49]. To do so, participants will be exposed to an increasing and progressive hierarchy of 5 different standard virtual cocaine-related situations, ordered according to the craving level anticipated by the participant for each situation. All the virtual cocaine-related situations will occur in a typical Martinican surrounding and housing (Fig. 3). Exposures to both proximal and distal cocaine-related cues will occur based on the following scenario: peers talk about cocaine (situation 1; Fig. 4) while using it (situation 2; Fig. 5), then the participant gets offered cocaine and virtually holds cocaine as well as its related paraphernalia (e.g., steel spoon, paper tubes, glass pipes, or syringes; situation 3; Fig. 6), thus prepares (situation 4; Fig. 7) and self-administrates (i.e., snorting, smoking or injecting; situation 5; Fig. 8) cocaine doses placed in front of him while seated at a table. For acceptability reasons, participants will switch to another VRCET environment once the self-reported cocaine craving level decreases by 50% for 5 continuous VRCE min (must be ≤ 3/10; 0 = none; 10 = very high).

Moreover, for ecological validity purposes, virtual cocaine paraphernalia, doses, and self-administration will be individualized depending on whether the participant’s main use consists of snorting, smoking, or injecting cocaine/crack. Additionally, audio dialogs from virtual avatars are adapted to both Martinican Creole and metropolitan French speakers (speech script available on request). In addition, in order to increase participants embodiment in VR, i.e., the sense of ownership and agency over the virtual body [50], participant virtual skin color will be personalized on request. Finally, for safety and acceptability purposes, given long lasting carry-over effects that could be expected on substance craving and emotional states post cue-exposure [51], VRCEs will have a 10-min washout period, and VRCET sessions will systematically end with a cool-down relaxing procedure with craving check [29, 52].

##### Memory-focused cognitive therapy—week 3

Following the 2-week VRCET phase, participants involved in the VRCET + experimental arm will receive an intensive 1-week MFCT phase delivered daily from Monday to Friday. The MFCT will be structured in accordance with the “Memory Focused Cognitive Therapy for Cocaine Use Disorder” Therapist Guide [39]. MFCT meetings will essentially consist of 5 sequential components: (1) Cognitive case conceptualization of CUD maintaining processes; (2) Education about cocaine’s cognitive and physical effects; (3) Cocaine-related cue-induction to elicit images and affective responses; (4) Memory reconsolidation procedures; (5) Standard CBT techniques (e.g., behavioral experiments of cocaine-related expectancies and skills for adaptive emotion regulation).

#### Active arm (PCET)

##### Picture-based cue-exposure therapy (PCET)—weeks 1 to 3

The active control arm will consist of an intensive 3-week treatment of 15 sessions of PCET. The PCET phase will consist of a total of 60 repeated 10-min PCE to cocaine-related cues spread out on 15 PCET sessions. All meetings will last 90 min. PCET sessions will occur during 3 consecutive weeks period (weeks 1, 2, and 3) and delivered on 5 consecutive days—from Monday to Friday. As aforementioned and outlined for VRCET, PCET will be habituation-focused only.

PCET will be performed using laptop-displayed standard PowerPoint slides stemming from VRCE screenshots (Figs. 4, 5, 6, 7, and 8). Participants will be seated on a stool in front of the laptop and PCE will be non-interactive. PCE structure, situations, hierarchy, and switching rules from one PCE situation to another will be similar to the ones outlined for VRCET. See Table 1, Table 2, and Table 3 for detailed descriptions of VRCET, PCET, and MFCT.

**Table 2:** Structure of CET sessions across VRCET and PCET interventions (experimental and active control arms)

**Welcoming participant** Active listening and problem solving on between-sessions cognitive-emotional difficulties; Resuming CET session plan and principles
**VRCE/PCE #1:** Situation 3 [10 min]
**Cocaine craving measures** During cue exposure (orally assessed): single-item (/10) Immediately after cue exposure (self-reported questionnaire): CEQ (S)
Wash-out procedure: resuming cue exposure course
**VRCE/PCE #2:** Situation 1 (10 min)
**Cocaine craving measures** During cue exposure (orally assessed): single-item (/10) Immediately after cue exposure (questionnaire): CEQ (S)
Wash-out procedure: resuming cue exposure course
**VRCE/PCE #3:** Situation 3 (10 min)
**Cocaine craving measures** During cue-exposure (orally assessed): single-item (/10) Immediately after cue exposure (questionnaire): CEQ (S)
Wash-out procedure: resuming cue-exposure course
**VRCE/PCE #4:** Situation 1 (10 min)
**Cocaine craving measures** During cue exposure (orally assessed): single-item (/10) Immediately after cue exposure (questionnaire): CEQ (S)
**Wash-out and safety procedure**: resuming cue-exposure course and session; paced-breathing relaxation; cocaine craving check
**End of CET session**

^*^During the next CET session, for cue exposure between/intra-session spacing and diversifying purposes, participants will be exposed to the ensuing situations order, according to their individualized exposures hierarchy: Situation 4, Situation 2, Situation 4, Situation 2

**Table 3:** Number and content of MFCT sessions (experimental arm; [39])

	**Session content**
Session # 1	•Resuming MFCT plan
	•Psychoeducation about cocaine effects on brain
	•Advantages and disadvantages of quitting cocaine use
	•Functional analysis of recent using episodes (situational, cognitive and emotional triggers)
Session # 2	•Psychoeducation about memory reconsolidation
	•Functional analysis of recent cocaine use episodes (situational, cognitive and emotional triggers)
Session # 3	•Relieving of one significant cocaine use episode
	•Out of relieving-cognitive restructure
Session # 4	•Relieving of one significant cocaine use episode
	•In relieving-cognitive restructure
Session # 5	•Relieving of one significant cognitively-restructured cocaine use episode
	•Relapse prevention focused-skills training

### Criteria for discontinuing or modifying allocated interventions {11b}

Participants will be able to discontinue their participation any time during the protocol. The principal investigator will oversee documenting reasons of early cessations thoroughly. The principal investigator will be allowed to permanently end any participation in the study, for any reason putting at significant risk participant safety or compliance to the protocol. Criteria for discontinuation include:

- Long-term hospitalization (> 1 month) for other than CUD
- Absence to 3 consecutive sessions

In case of discontinuation, the data collected will be analyzed and the participants will not be substituted.

### Strategies to improve adherence to interventions {11c}

Standardized training and scripted therapy handbooks (available on request) will be provided to the facilitators to ensure therapy adherence and reliable delivery of the experimental and active control treatments, and to make further replication possible.

### Relevant concomitant care permitted or prohibited during the trial {11d}

Participants involved in concomitant treatment as usual (TAU) psychotherapeutic or pharmacologic treatments for CUD other than those provided in the inpatient setting will be maintained in the protocol, but their data will be excluded for comparability purposes.

### Provisions for post-trial care {30}

The University Hospital of Martinique (UHM), this trial’s sponsor, has contracted an insurance cover upon the Lloyd’s Insurance Company (ID: MCIEEA21042) for its public liability as well as that of any person involved in the trial, which is in accordance with the French public health code.

### Outcomes {12}

**Fig. 9:**
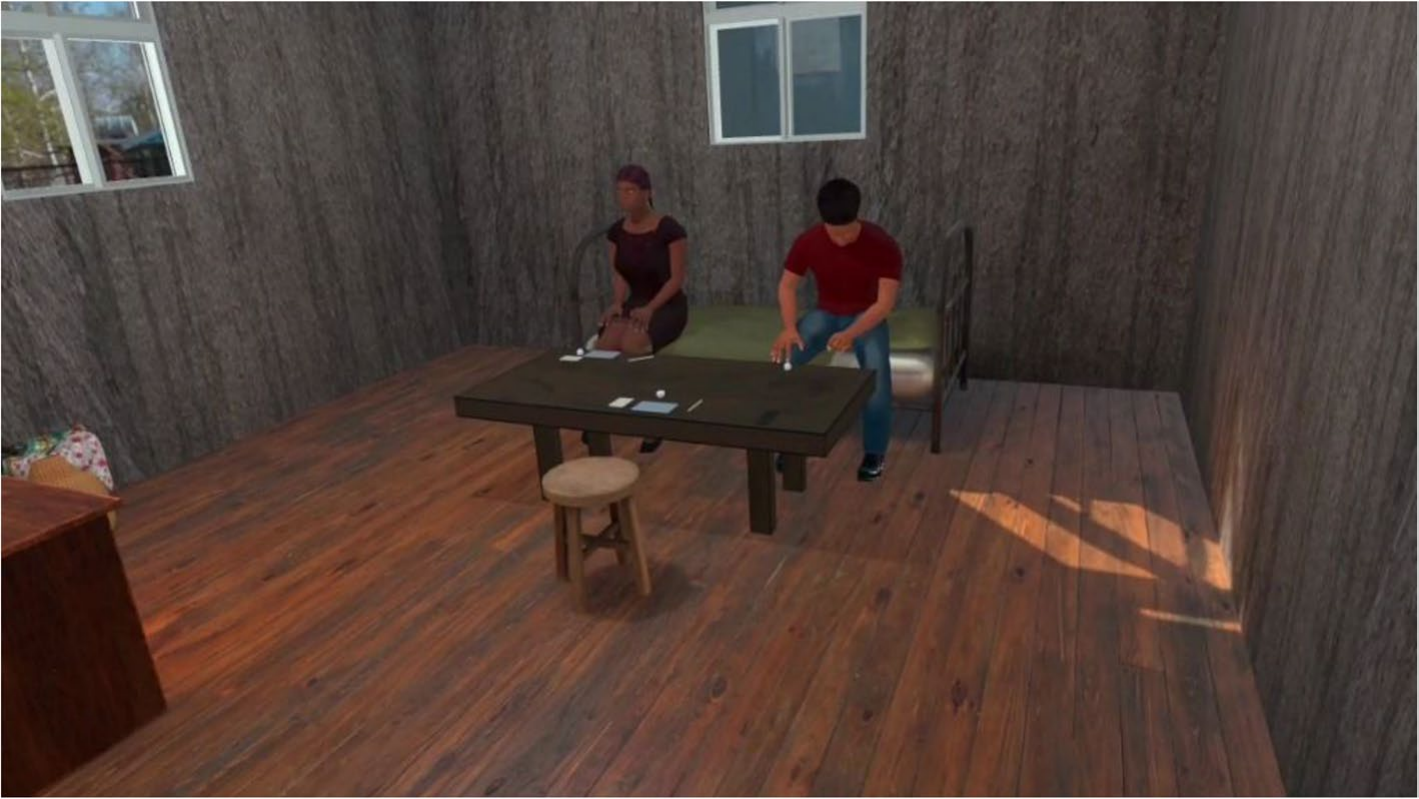
Virtual 1st-person view from VRCE or PCE in the craving induction task

Our primary outcome will consist in the decrease of the self-reported cocaine craving as assessed with the French Craving Experience Questionnaire (CEQ; [53, 54]. The CEQ is an 11-item retrospective measure of cocaine craving containing two subscales: craving strength (CEQ-S) and frequency (CEQ-F). Each item is completed by the participants on a 10-point scale, from “not at all” (1) to “extremely” (10). The CEQ has shown good psychometric properties [54]. Means of CEQ-S and CEQ-F total scores will be analyzed. Finally, the CEQ will be administered before treatment (V0), after 2 weeks of treatment (V1; VRCET vs. PCET), post-treatment (V2; VRCET + vs. PCET), and 1-month post-treatment (V3; VRCET + vs. CET). The CEQ-S will be administrated immediately after a 7-min VRE to a cocaine-related situation, with a similar scenario to the CET situation 5 (Table 1) but across different virtual environments, avatars, and item appearances (Fig. 9). The CEQ-F will be administrated retrospectively, targeting the last 14-day period. (Table 4)

**Table 4:** Schedule for the PICOC study

		**Study period**			
	**Enrolment and allocation**	**Post-allocation**			**1-month follow-up**
**TIMEPOINT**	**T0**	**T1**		**T2**	**T3**
	**Week 0**	**Week 1**	**Week 2**	**Week 3**	**Week 7**
**ENROLMENT:**					
**Eligibility screen**	X				
**Informed consent**	X				
**Allocation**	X				
**INTERVENTIONS:**					
**Experimental group**					
**VRCET MFCT**		--------------------------			
				-----------	
**Active control group**					
**PCET**		--------------------------			
**ASSESSMENTS:**					
**CEQ-F, TLFB, DERS, AWARE, QCBDU ** ^**a**^	X		X	X	X
**In cue exposure (CET only):** **CEQ-S, orally assessed cocaine craving, SPIE** ^**b**^ **, ITC-SOPI** ^**c**^		X	X	X	
**In VRE to cocaine cues:** **CEQ-S, BMIS, DTCQ-8D, blood pulse, skin temperature and conductance**	X		X	X	X
**Cocaine urine test strip**	X		X	X	X
**CSQ-8**			X	X	

^a^ For each new exposure situation encountered; ^b^ ITC-SOPI Ecological validity subscale; ^c^ QCBDU – Beliefs Related to Addiction subscale

Secondary outcomes will consist of:

1. Assessing the effects after 2 weeks of treatment (V1; VRCET vs. PCET), post-treatment (V2; VRCET + vs. PCET), and 1-month post-treatment (V3; VRCET + vs. PCET) on cocaine use-related measures (abstinence, risk of relapse, emotional dysregulation, and dysfunctional thoughts) measured via urinary samples and self-reported questionnaires.
  Negative cocaine urine test strips will be used for objectively support participant self-reports of cocaine abstinence [55].
  Timeline Followback (TLFB) mean total scores will be used for subjectively assess participants cocaine abstinence in within the last 21 days, ranging from 0 (“none”) to 100 (“total abstinence”; [56]).
  Advance Warning of Relapse (AWARE) mean total scores will be used to subjectively assess the decrease of participants risk of cocaine use relapse within the next 2 months, ranging from 25 (11 to 37%-risk) to 193 (53 to + 95%-risk; [57]).
  The Difficulties in Emotion Regulation Scale (DERS) mean total scores will be used to subjectively assess the decrease of participant emotional dysregulation, ranging from 36 (“Almost never”) to 180 (“Almost always”; [58]).
  The Questionnaire of Core Beliefs related to Drug Use and Craving (QCBDU) – Beliefs Related to Addiction mean total scores will be used to subjectively assess the decrease of participant identification to dysfunctional thoughts related to CUD, ranging from 0 (“Strongly disagree”) to 72 (“Strongly agree”; [59]).
2. Assessing at V0 the effects of VRCE to cocaine cues on cue-specific cocaine craving-related subjective (cocaine craving, emotional states, self-efficacy to cope with craving), physiological reactivity (blood pulse, skin temperature and conductance), and cue-exposure specific measures (sense of presence and ecological validity), as compared with PCE. The physiological measures and the self-reported measures will be administered, respectively, during and immediately after a 7-min VRE or PCE to a cocaine-related situation, with similar scenario to the CET situation 5 (Table 1), but with different virtual environments, avatar, and item appearances (Fig. 9).
  The dispersion of blood pulse, skin temperature, and conductance signals will be used through PLUX Biosignals physiological captors (medical device certification: ISO 13485) to assess the cue-specific reactivity in VRCE compared to PCE.
  The CEQ-S mean total scores will be used to subjectively assess cocaine craving intensity in participants exposed to VRCE compared to PCE; items range from 11 (“not at all”) to 110 (“extremely”; [54]).
  The Brief Mood Introspection Scale (BMIS) mean total scores will be used to subjectively assess negative and positive emotional states in participants exposed to VRCE compared to PCE. Items range from 0 (“definitely do not feel”) to 21 (“definitely feel”; negative emotions) or 27 (positive emotions; [60]).
  The Drug Taking Confidence Questionnaire – 8 (DTCQ-8) mean total scores will be used to subjectively assess the self-efficacy to cope with cocaine craving in participants exposed to VRCE compared with PCE; items range from 0 (“not at all confident”) to 800 (“very confident”; [61]).
  The Spatial Presence for Immersive Environments (SPIE) – Spatial Presence subscale mean total scores will be used to subjectively assess the sense of presence experienced by participants exposed to VRCE compared with PCE; items range from 4 (“strongly disagree”) to 20 (“strongly agree”; [62].
  The Spatial Presence for Immersive Environments (SPIE) – Realism subscale mean total scores will be used to subjectively assess the increased sense of reality experienced by participants exposed to VRCE compared with PCE, ranging from 4 (“strongly disagree”) to 15 (“strongly agree”; [62]).
  The Independent Television Commission Sense of Presence Inventory (ITC-SOPI) – Ecological Validity subscale mean total scores will be used to subjectively assess the increased sense of ecological validity experienced by participants exposed to VRCE compared to PCE; items range from 5 (“strongly disagree”) to 25 (“strongly agree”; [25]).
3. Assessing the cognitive, behavioral, or emotional factors (cocaine craving, dysfunctional thoughts, cocaine use, and emotional dysregulation) mediating the efficacy of VRCET+ in decreasing self-reported cocaine craving. The CEQ – F, QCBDU, TLFB, and DERS mean total scores will be used to subjectively assess, as aforementioned, cocaine craving frequency (last 14 days), dysfunctional thoughts regarding CUD, cocaine abstinence (last 21 days), and difficulties in emotion regulation.
4. Assessing the acceptability of (i) our intensive VRCET as a stand-alone (after 2 weeks of treatment; V1) or add-on (post-treatment; V2; VRCET +) intervention and (ii) VRE as a cue-exposure paradigm, for each new VRE performed in therapy.
  The Spatial Presence for Immersive Environments (SPIE) – Cybersickness subscale mean total scores will be used to subjectively assess acceptable cybersickness symptoms (i.e., < 7) experienced by participants exposed to VRCE; items range from 0 (“strongly disagree”) to 10 (“strongly agree”; [62]).
  The Client Satisfaction Questionnaire – 8 (CSQ-8) mean total scores will be used to subjectively assess satisfaction levels towards psychotherapeutic services (≥ 21; “good”); items range from 8 to 32 [63].

### Participant timeline {13}

#### Inclusion visit (V0)

Every patient treated for CUD at one of the two investigations centers and deemed potentially eligible for study inclusion will be informed by their assigned psychiatrist about the study’s objectives, protocol implications, and expected therapeutic benefits. Each patient will be invited to the inclusion visit (V0) during which the necessary information for giving informed consent to participate will be provided. The dates of the written consent as well as its withdrawn (if applicable) will be documented in their medical record. During this inclusion visit, the research psychiatrist or psychologist will oversee the examination of study eligibility for patients (e.g., Significant SCID-5 CV score for CUD; URICA self-questionnaire score ≥ 11; Non-significant M.I.N.I.5. (DSM-IV) score for current high suicidal risk, post-traumatic stress, psychotic, mania or hypomania episode; Post-VRE SPIE—Cybersickness score ≥ 7).

#### Randomization

Eligible patients will be randomized by the research psychiatrist or psychologist in charge of the inclusion visit, using the Research Electronic Data Capture (REDCap) secure web application, in the VRCET + (experimental) or PCET (active control) therapeutic arm.

After randomization, participants will be invited to complete baseline self-reported measures as well as to perform a craving induction task related to primary and secondary research objectives. This craving induction task will consist of three 7-min 30 exposures and will systematically start with one VRE to neutral cues, followed by one VRE and one PCE to a cocaine-related situation. The order of these two exposures to cocaine cues (i.e., VRE thus PCE or PCE thus VRE) will be randomly counterbalanced for controlling for potential cue-induced carry-over effect between one exposure to the other. The exposures’ order sequence generation for each participant will be generated by the research methodologist, using the Research Randomizer secure web application, and provided to the investigator within sealed opaque envelope for the inclusion visit.

#### Experimental or active control therapy (3 weeks)

Participants included will start therapy (experimental or active control arm) within 1 week after the inclusion visit as follows:

- VRCET + (experimental arm): 2-week phase of 10 VRCET sessions, followed by a 1-week phase of 5 MFCT sessions. The VRCET phase will essentially consist of 40 VRE to a progressive hierarchy of 5 diversified cocaine-related situations. The MFCT phase will be in accordance to the “Memory Focused Cognitive Therapy for Cocaine Use Disorder” therapist guide (for request: [39]), and will consist of cognitive restructuring and memory reconsolidation procedures along with imagery exposure to arousing memories of cocaine use.
- PCET (active control arm): 3-week phase of 15 PCET sessions. The PCET phase will essentially consist of 60 VRE to a progressive hierarchy of 5 diversified cocaine-related situations.

Both VRCET + and PCET sessions will last 1 h 30 min and will be delivered in an intensive 1:1 intervention format, on a daily basis, from Monday to Friday. The facilitator of VRCET + and PCET may differ, but each patient will have only one facilitator.

#### In and post-treatment measuring times (V1, V2, and V3)

The first measuring time (T1) will take place after 2 weeks of treatment, at the 11th treatment session and prior to start the MFCT (experimental arm) or second PCET (active control arm) phase. This will thus allow for comparing short-term research primary and secondary outcomes between VRCET and PCET, after 10 consecutive sessions of exposure.

The second measurement time (T2) will take place at the end of treatment (week 3), at the 15th and last treatment session. This will thus allow for comparing short-term research primary and secondary outcomes between VRCET + (10-session VRCET and 5-session MFCT) and PCET, after 15 consecutive sessions.

The third and last measuring time (T3) will consist in a follow-up visit taking place 1 month after the end of treatment (week 8). This will thus allow for comparing medium-term research primary and secondary outcomes between VRCET + (10-session VRCET and 5-session MFCT) and PCET, 1 month after 15 consecutive sessions of each therapeutic arm. Patients’ participation will end after this visit.

**Fig. 10:**
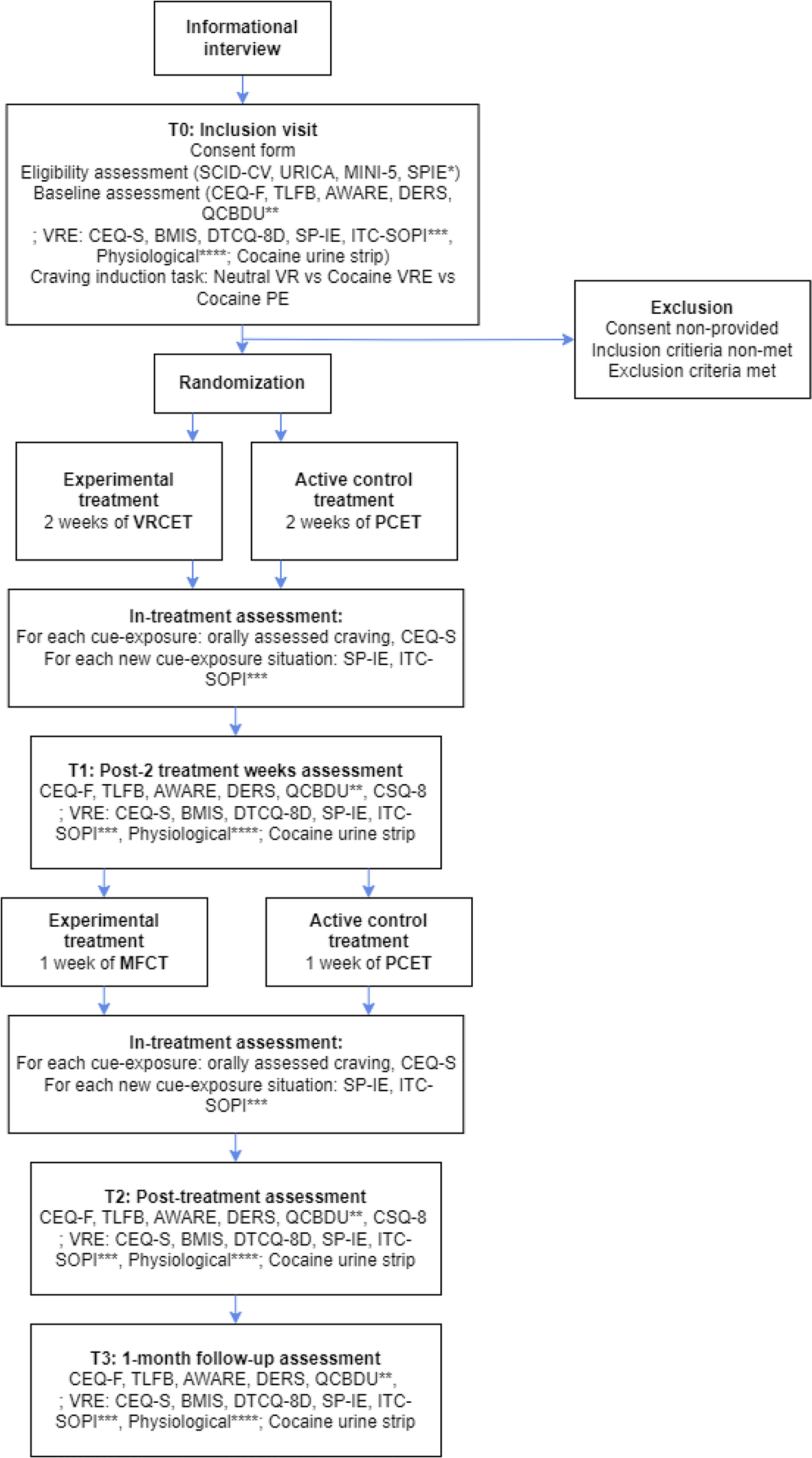
Flow chart for the PICOC study

For each of these measurement times, subjective self-report questionnaires and objectives measures related to research objectives will be administered (Fig. 10).

### Sample size {14}

We calculated a required sample size of 54 participants to randomize with G*Power [64], on the basis of two-tailed independent *t*-tests, to detect large effect from our experimental treatment (VRCET +) over our control therapeutic condition (PCET) in reducing cocaine craving [39, 65], given a 0.05 statistical significance threshold, a 0.95 statistical power, and accounting for potential dropouts in our hospitalized inpatients.

### Recruitment {15}

We will benefit from a significant network of psychiatrists, psychologists, and addictology and health professional workers from the University Hospital of Martinique (UHM) and the Saint-Esprit Hospital (SEH; Martinique) to steer any patient volunteering to participate in the study. To date, both UHM and SEH inpatient residential addictology clinic provide access to 30 beds for TAU for CUD, reaching up to 200 patients per year.

## Assignment of interventions: allocation

### Sequence generation {16a}

The Research Electronic Data Capture (REDCap) secure web application will be used for randomizing participant allocation to the experimental or active control therapeutic arm. The randomization will be stratified per investigation center (UHM or SEH), with an allocation ratio of 1:1 to the therapeutic arms and following a randomly permuted per-block order.

### Concealment mechanism {16b}

The research psychiatrist or psychologist will be informed of the participant allocation only at the start of the intervention phase, following a sealed opaque envelope-based procedure.

### Implementation {16c}

The allocation sequence will be generated by the research data manager from the UHM methodology unit before starting the research and following a confidential handbook document. Each research psychiatrist and psychologist will oversee the assignment of participants to interventions in their respective investigation center.

## Assignment of interventions: blinding

### Who will be blinded {17a}

Patients and researchers will not be blinded after assignment to interventions given significant delivery modalities between VR-based and picture-based exposure therapies. Only the data manager in charge of the allocation sequence will be blinded to the assignment.

### Procedure for unblinding if needed {17b}

No unblinding procedure will occur given our trial design open-label nature.

## Data collection and management

### Plans for assessment and collection of outcomes {18a}

Study outcomes will be mostly assessed and collected using paper-based self-report questionnaires. Reliability or medical certification of these psychometrically validated self-report and physiological instruments are detailed in the “Outcomes {12}”. The study assessors will be trained for assessing and collecting outcomes prior to the research. Patient individual data will be recorded on an online hospital server using the Research Electronic Data Capture (REDCap) secure web application.

### Plans to promote participant retention and complete follow-up {18b}

Prior to each measurement time (T0, T1, T2, and T3), participants will be reminded of the therapeutic and scientific importance of dully completing each intervention and assessment phase. For the follow-up assessment, the investigators will contact the participants to collect study outcomes. A 20 € voucher will be offered to any outpatient participant in the follow-up assessment phase willing to use public transport for coming to their respective investigation center. Participants will be reminded that they may discontinue their consent to participate at any time during protocol, without justifying any reason and with no impact on their TAU. Data collected in participants who withdraw their consent will be analyzed.

### Data management {19}

For each patient included on the trial, an e-CRF will be generated and dully completed with participants’ individual data collected along the protocol. Access to this e-CRF will be granted through the Research Electronic Data Capture (REDCap) secure web application, with rights (e.g., data visualization and modification), identification, and password sequences specific to each study member. Data entry will be secured using a crypted 128 bits SSL mode directly on internet navigator and recorded (data provider, data provided, and date of data entry) following an audit trail system.

### Confidentiality {27}

Any study professional member (e.g., research psychiatrist, psychologist, or data manager) having access to participants’ individual data will be bound to professional secrecy, which is in accordance with the French public health code. The use of participants’ individual data collected will be systematically anonymized following a codification participant identification procedure. To do so, each code generated for each participant will include the center in which the investigation is conducted (UHM or SEH), the intervention assignment (experimental or active control arm), and the inclusion number (e.g., 001), following a chronologic sequence. Each principal investigator will oversee the confidentiality of the data using a sealed paper-based record in which participant authentication data (first name, family name, and date of birth) will be related to their identification code.

### Plans for collection, laboratory evaluation and storage of biological specimens for genetic or molecular analysis in this trial/future use {33}

N/A: no collection, laboratory evaluation, and storage of any biological specimens for genetic or molecular analysis in this trial/future use.

## Statistical methods

### Statistical methods for primary and secondary outcomes {20a}

Statistical analyses for principal and secondary outcome will be computed from self-report scales raw total scores or physiological signals (i.e., blood pressure, skin temperature and conductance) using, respectively, R Statistical [66] and OpenSignals (© PLUX S.A.) software. Our comprehensive data working R code, including R packages and inferential statistical hypothesis testing assumptions (Gaussian distribution, homoscedasticity, etc.), will be made available. For each primary or secondary variable of interest, descriptive statistics (n, min, max, median, q1, q3, iqr, mean, sd, se, and mean ci) will be computed using the *rstatix R* package. Primary inferential statistical analyses will consist of two-tailored independent *t*-tests on score changes between T0 (baseline) and T2 (post-treatment) in the experimental (i.e., VRCET +), compared with the active control therapeutic arm (PCET). In case of mostly non-normal variable distributions but symmetrical score differences around the median, Friedman and Bonferroni-corrected Wilcoxon signed-rank tests will be performed to test and contrast score differences between the conditions using *stats* and *rstatix R* packages [67]. In case of non-symmetrical paired score differences, Bonferroni-corrected Sign tests will be performed. Causal mediation analyses will be adjusted for confounding factors. Cohen’s d, Kendall’s W, and Cliff’s delta effect sizes for score changes across treatment arms (“negligible,” “small,” “medium,” and “large” effect for Cliff’s d inferior to 0.15, 0.33, 0.47, and superior to 0.47; [68]) with 95% CIs will be computed using *irr* and *effsize R* packages.

### Interim analyses {21b}

N/A: No interim analyses are planned.

### Methods for additional analyses (e.g., subgroup analyses) {20b}

N/A: No subgroup analyses are planned.

### Methods in analysis to handle protocol non-adherence and any statistical methods to handle missing data {20c}

Given the small target sample size (*n* ≈ 50) and in case of arbitrary pattern of missing data, if applicable, multiple imputations for incomplete multivariate data will be performed on all missing data, using the *Amelia R* package [69, 70]. Our comprehensive imputation *R* code will be made available.

### Plans to give access to the full protocol, participant-level data, and statistical code {31c}

Any full-protocol-related information or material (e.g., consent form or therapy guide), statistical code, and participant-level dataset will be made available upon request.

## Oversight and monitoring

### Composition of the coordinating center and trial steering committee {5d}

Two investigators will oversee the supervision of the overall research trial in their investigation center. Another co-coordinator investigator will supervise the trial opening and assisting its course (e.g., handbook psychotherapy implementation and adherence) on its early phase. A steering committee, composed of each research investigator, the research scientific committee, the research methodologist and a promoter agent designed the research, wrote the protocol and will be in charge of deciding about the trial update and continuity.

### Composition of the data monitoring committee, its role and reporting structure {21a}

Given our trial design open-label nature, no data monitoring committee will be required to protect blinding of the research psychiatrists and psychologists. Two data managers will be in charge of managing, assessing data entry quality, and providing access to the Research Electronic Data Capture (REDCap) secure web application.

### Adverse event reporting and harms {22}

According to article L1123-10 of the French code of public health, any adverse reaction/incident will be reported through the Ministry of health’s adverse health event reporting portal. Minor adverse events such as fatigue or hypersomnia due to protocol compliance and cocaine abstinence are anticipated. No serious adverse events related to the protocol are anticipated. As soon as the participant consent form is collected and until its participation ending, the trial principal investigator will also inform the promoter and take note of any spontaneous significant participant adverse health event deemed potentially related to the research protocol into the Research Electronic Data Capture (REDCap) secure web application.

### Frequency and plans for auditing trial conduct {23}

At any stage of the trial, a clinical and independent research associate, designed by the study promoter, will be allowed to audit, and inspect any study member, document, procedure, or data related to the trial conduct in order to guarantee the well respect of participant safety, rights, and data integrity. This inspection can also be solicited from another competent authority, such as the French national agency for the safety of medicines and health products (ANSM). In any case, the investigators will comply with the inquiries from the research sponsor or any other competent authority conducting the audit or the inspection.

### Plans for communicating important protocol amendments to relevant parties (e.g., trial participants, ethical committees) {25}

Any substantial modification brought to the protocol, i.e., at significant risk for participant safety, research result validity, adherence to the planned therapeutic arms, will be addressed under written form to the research sponsor, upon approbation from the French research ethics committee (*Comité de Protection des Personnes – Île-de-France VII*). Other non-substantial modifications will be noticed to the research ethic committee for information purpose. Any modification, including those at risk of plausible incidence on participant benefits or constraints, will be updated to the trial register (i.e., clinicaltrials.gov: NCT05833529).

### Dissemination plans {31a}

Findings of this trial will be disseminated to psychiatry and addictology-related international and national conferences, as well as manuscript publications to peer-reviewed journals. A results summary will be made accessible to study participants and updated to the trial register.

## Discussion

To the best of our knowledge, our project is the first randomized and controlled trial of VRCET for CUD, primarily assessing its efficacy and acceptability as a therapeutic add-on (i.e., combined with MFCT) to reduce both cocaine use frequency and intensity post-intervention (T2) and at 1-month follow-up (T3), as compared with an active control therapy (PCET). Therapeutic evidence on VRCET integrated in broader treatment for SUD are mixed [30]. For instance, a randomized and controlled trial (RCT) in 44 patients hospitalized for alcohol use disorder (AUD) reported a significant decrease of cue-induced self-reported craving and physiological reactivity in an experimental arm combining VRCET with conventional treatment for SUD, compared to conventional treatment [71]. Another RCT in 102 patients with NUD did not observe significant differences in retention and abstinence rates between a CBT + CET experimental arm and a CBT control arm up to 12-month follow-up [36]. Numerous other CET trials in the literature combine habituation-focused interventions with miscellaneous CBT techniques (e.g., coping skills training) into one single exposure session [30]. While authors argue for CET-specific (e.g., increased habituation) and non-specific factors (e.g., reduced substance intake) for explaining cue-induced craving reduction [36, 72], any causal attribution of changes related to VRCET or any other psychotherapeutic condition remain highly limited without a proper comparative design, as intended in our trial [30, 45]. In addition, our CET sessions (VRCET and PCET) will be exclusively habituation-focused, i.e., implying a decrement in cue-induced craving response after its repeated, prolonged, and non-reinforced stimulation [47, 48]. Taken together, our CET comparative design is assumed to bear high internal validity which is a prerequisite for inferring about reliable habituation-based mechanisms of change between VRE and PCE, (i.e., respectively, the experimental and the active control arm).

Further, while comparable to superior effects of VRE over classical exposure methods on craving induction in SUDs have been reported [73, 74], to the best of our knowledge, no study has assessed the specific interest of VRCE over a non-VR cue-exposure approach in a CET context [27]. Our trial design will allow to fill the gaps in the literature by specifically assessing the effects of VR, both as a cue-reactivity exposure and cue-exposure therapy paradigm, on cocaine craving in patients with CUD. Indeed, the effects of VR as a cue-reactivity paradigm (i.e., VRE) on cocaine craving-related reactivity and exposure-dependent characteristics (i.e., sense of presence, ecological validity and cybersickness) will be contrasted to the ones of a non-VR cue-reactivity frame (i.e., pictures-based; PCE) at baseline (T0). On the other hand, the acute benefits of VRCET as a stand-alone CET will be compared to the ones a non-VR cue-exposure therapy frame (i.e., PCET) after 2 weeks of a 10-session treatment (T1). We expect to obtain superior effects of VRE or VRCET over PCE and PCET on craving induction and reduction. This may be explained by the fact that VRE environments are designed to be highly immersive (head-mounted display), interactive, multisensorial and implying 3D environments that can include diversified proximal and distal stimuli adapted to the Martinican participants ecological substance using field [21]. Hence, we hypothesize that higher self-reported sense of presence, ecological validity, and realism levels will be self-reported by participants exposed in our high-immersive VRE, as compared with in PCE. Such findings are consistent with meta-analytic evidence (*N* = 115 studies) showing the significant and medium effect of VR immersive features on sense of presence (*r* = 0.316; [75]). In addition, cue-reactivity studies in smokers and heavy drinkers suggest that sense of presence, ecological validity, and realism in VRE significantly predict [76, 77] and enhance craving induction [26]. Furthermore, there is a consensus on the fact that VRCET, compared to PCET, allows diversified exposure to motor, proprioceptive and 3D cues related with cocaine use that could not be used in vivo for safety purposes [21]. Hence, we believe that, compared to PCE, VRCE will allow for broader generalization of CET benefits (i.e., craving reduction) over the desensitization context, and prevent cue-induced craving rebound (e.g., renewal effect) in CUD [22, 23]. Finally, acceptability studies of VRCET as a stand-alone intervention or adjunct to regular treatment for SUD remain scarce [78–80]. In accordance with the findings by Arissen et al. [78], Skeva et al. [79], and Wray and Emery [80], to guarantee the acceptability of VRCE and VRCET, we focused on designing low-cybersickness VR interfaces, shortening and spacing VRCE sessions, and familiarizing therapy facilitators and patients with VRCET and VR prior to the therapeutic phase.

To conclude, a growing body of evidence suggests that enhancing cue-exposure might be of promising value for clinical addictology since craving response or reactivity to smoking cues significantly moderates craving reduction [18] and predicts drinking latency, dependence severity and withdrawal reinstatement in a CET context [19]. To do so, VR appears as a promising tool to decrease craving, notably in CUD whereby it has been understudied. Our RCT aims at filling the gaps in the literature and investigate the clinical efficacy and acceptability of VRCET over non-VR CET or CBT for CUD [27].

## Trial status

Study recruitment began in May (01st) 2023. Recruitment is estimated to be completed in May 2025 (31th). This version is the first of the protocol.

## Data Availability

This study protocol has been reported according to the Standard Protocol Items: Recommendations for Clinical Interventional Trials (SPIRIT) guidelines. Any protocol related information or material, statistical code and dataset will be made available upon request.

## Abbreviations

AWARE: Advance Warning of Relapse questionnaire
BMIS: Brief Mood Introspection Scale
CEQ – F: Craving Experience Questionnaire – Frequency subscale
CEQ – S: Craving Experience Questionnaire – Strength subscale
CSQ-8: Client Satisfaction Questionnaire – 8-item version
CBT: Cognitive Behavioral Therapy
CET: Cue-exposure therapy
CUD: Cocaine use disorder
DERS: Difficulties in Emotion Regulation Scale
DSM-IV: Diagnostic and Statistical Manual of Mental Disorders – 4th version
DTCQ-8: Drug Taking Confidence Questionnaire – 8-item version
ITC-SOPI: Independent Television Commission – Sense of Presence Inventory
MFCT: Memory-focused cognitive therapy
M.I.N.I.5.: Mini Internation Neuropsychiatric Interview – 5th version
PCE: Pictures-based cue-exposure
PCET: Pictures-based cue-exposure therapy
QCBDU: Questionnaire of Core Beliefs related to Drug Use and craving – Beliefs Related to Addiction subscale
SCID-5 CV: Structured Clinical Interview for DSM-5 ® disorders—Clinical version
SPIE: Spatial Presence for Immersive Environments questionnaire
SUD: Substance use disorder
TLFB: Timeline Followback questionnaire
URICA: University of Rhone Island Change Assessment Scale
VR: Virtual reality
VRCE: Virtual reality-based cue-exposure
VRCET: Virtual reality cue-exposure therapy
VRCET +: Virtual reality cue-exposure therapy + memory-focused cognitive therapy
VRE: Virtual reality-based exposure
3D: Three dimensional
